# First External Quality Assessment of Molecular and Serological Detection of Rift Valley Fever in the Western Mediterranean Region

**DOI:** 10.1371/journal.pone.0142129

**Published:** 2015-11-13

**Authors:** Federica Monaco, Gian Mario Cosseddu, Baba Doumbia, Hafsa Madani, Fatiha El Mellouli, Miguel Angel Jiménez-Clavero, Soufien Sghaier, Philippe Marianneau, Catherine Cetre-Sossah, Andrea Polci, Sandra Lacote, Magtouf Lakhdar, Jovita Fernandez-Pinero, Chabane Sari Nassim, Chiara Pinoni, Andrea Capobianco Dondona, Carmina Gallardo, Taoufiq Bouzid, Annamaria Conte, Grazia Bortone, Giovanni Savini, Antonio Petrini, Lilian Puech

**Affiliations:** 1 Istituto Zooprofilattico Sperimentale dell’Abruzzo e del Molise “G. Caporale”, Teramo, Italy; 2 Centre National d'Elevage et de Recherches Vétérinaires, Nouakchott, Mauritania; 3 Institut National de la Médecine Vétérinaire, Laboratoire Central Vétérinaire d'Alger, Alger, Algeria; 4 Office national de sécurité sanitaire des produits alimentaires (ONSSA), Laboratoire Régional d'Analyses et de Recherches de Casablanca, Casablanca, Morocco; 5 Centro de Investigación en Sanidad Animal (CISA-INIA), Valdeolmos, (Madrid), Spain; 6 Institut de Recherche Vétérinaire de Tunisie, Université Tunis El Manar, Tunis, Tunisia; 7 Agence nationale de sécurité sanitaire de l’alimentation, de l’environnement et du travail (ANSES), Lyon, France; 8 Centre de Coopération Internationale en Recherche Agronomique pour le Développement (CIRAD), UMR CMAEE, Institut National de la Recherche Agronomique (INRA), UMR 1309, F-34398 Montpellier, France; 9 Institut National de la Médecine Vétérinaire, Laboratoire Vétérinaire Régional de Laghouat, Laghouat, Algeria; 10 Institut National de la Médecine Vétérinaire, Laboratoire Vétérinaire Régional de Tlemcen, Tlemcen, Algeria; 11 Office national de sécurité sanitaire des produits alimentaires (ONSSA), Laboratoire Régional d'Analyses et de Recherches d'Agadir, Agadir, Morocco; 12 Ministère des Affaires étrangères et du Développement international, Paris, France; The University of Texas Medical Branch, UNITED STATES

## Abstract

Rift Valley fever (RVF) is a mosquito-borne viral zoonosis which affects humans and a wide range of domestic and wild ruminants. The large spread of RVF in Africa and its potential to emerge beyond its geographic range requires the development of surveillance strategies to promptly detect the disease outbreaks in order to implement efficient control measures, which could prevent the widespread of the virus to humans. The Animal Health Mediterranean Network (REMESA) linking some Northern African countries as Algeria, Egypt, Libya, Mauritania, Morocco, Tunisia with Southern European ones as France, Italy, Portugal and Spain aims at improving the animal health in the Western Mediterranean Region since 2009. In this context, a first assessment of the diagnostic capacities of the laboratories involved in the RVF surveillance was performed. The first proficiency testing (external quality assessment—EQA) for the detection of the viral genome and antibodies of RVF virus (RVFV) was carried out from October 2013 to February 2014. Ten laboratories participated from 6 different countries (4 from North Africa and 2 from Europe). Six laboratories participated in the ring trial for both viral RNA and antibodies detection methods, while four laboratories participated exclusively in the antibodies detection ring trial. For the EQA targeting the viral RNA detection methods 5 out of 6 laboratories reported 100% of correct results. One laboratory misidentified 2 positive samples as negative and 3 positive samples as doubtful indicating a need for corrective actions. For the EQA targeting IgG and IgM antibodies methods 9 out of the 10 laboratories reported 100% of correct results, whilst one laboratory reported all correct results except one false-positive. These two ring trials provide evidence that most of the participating laboratories are capable to detect RVF antibodies and viral RNA thus recognizing RVF infection in affected ruminants with the diagnostic methods currently available.

## Introduction

Rift Valley fever (RVF) is a mosquito-borne viral zoonosis which affects humans and a wide range of vertebrate hosts causing severe economic losses in adult livestock (primarily sheep, goats and cattle). The Rift Valley fever virus (RVFV) belongs to the *Bunyaviridae* family and the *Phlebovirus* genus. Its genome consists of 3 single stranded RNA segments, the M (medium) and L (large) segments are of negative orientation whereas the S (small) segment has an ambisense polarity [1]. The virus is primarily transmitted by mosquitoes to and among animals. Known competent vectors belong to the genera *Aedes*, *Culex and Anopheles* [2]. Direct transmission through contact with infected tissue may occur and play an important role in human infection [2].

The virus was first identified in 1930 along the shores of Lake Naivasha in the greater Rift Valley of Kenya [3,4]. During the last decades RVFV caused large epidemics in many African countries as well as Madagascar and the Arabian Peninsula, causing severe economic losses in breeding of ruminants and hundreds of human deaths [5,6]. Over time, the virus shows little variation, with one known serotype [7].The wide spread of RVF in Africa requires the development of surveillance strategies to promptly detect the disease outbreaks in order to implement efficient control measures, which could prevent the spillover of the virus to humans. Therefore, accurate detection of RVFV in animals and mosquitoes is essential. According to the World Organization for Animal Health-OIE [8], diagnostic methods for RVFV include virus isolation, reverse-transcription polymerase chain reaction (RT-PCR), and serological tests. Isolation procedures are expensive, time-consuming and require high biocontainment facilities (biosafety level 3- BSL3). Therefore several molecular diagnostic tests based on RT-PCR targeting the different segments of the virus genome have been recently developed [9–12], allowing the rapid and sensitive detection of the virus genome. Among the serological tests, ELISA is the most widely used technique for IgM and IgG type antibodies detection. Different ELISA formats are commercially available and others are currently under development [13–18].Virus neutralization test (VNT) is the prescribed test for international trade enabling RVFV antibodies detection in the serum of a large range of animal species. However it is time-consuming and requires skilled personnel working in BSL3 facilities[8].

The performance of the different techniques applied to the RVF diagnosis may vary between laboratories. An external quality assessment (EQA) allows the laboratories to monitor the quality of their diagnosis, evaluate their capacities and, eventually, identify the possible weaknesses in order to put in place corrective actions.

An Animal Health Mediterranean Network (REMESA) linking six Northern African countries Algeria, Egypt, Libya, Mauritania, Morocco, Tunisia and four Southern European countries, France, Italy, Portugal and Spain was created in 2009 with the technical support of the OIE and the Food and Agriculture Organization of the United Nations (FAO). The main aim is to improve the animal health with a particular focus on prevention and control of transboundary animal diseases, including zoonoses, through capacity building, coordination and practices harmonization. Cyprus, Greece and Malta joined the network in 2013, Jordan and Lebanon in 2014. The Chief Veterinary Officers (CVOs) members of REMESA recognized RVF as a priority deserving specific attention. As part of the REMESA, a veterinary diagnostic laboratory based network (RELABSA) was established to strengthen the national laboratories scientific and technical level diagnostic capacities. Integrated and inter-operative surveillance efforts in the region would benefit from the harmonization of the diagnostic procedures as well as setting in quality assurance for member laboratories.

In September–October 2010, an unprecedented outbreak of RVF was reported in the northern Sahelian region of Mauritania after exceptionally heavy rainfall. At the end of December 2010, a total of 63 cases among humans, including 13 deaths, had been officially reported, but the true number is probably much higher due to the remoteness of the affected area. Positive test results were observed by real-time reverse transcription PCR and the virus was isolated from initially positive serological samples [19].

Therefore, the regional OIE/FAO coordination unit of REMESA (OIE Sub-Regional Representation for North Africa and FAO Sub-regional office for North Africa, both located in Tunis, Tunisia) proposed to the CVOs of REMESA the implementation of a dedicated project, including laboratory, epidemiology and entomology activities. A specific financial support was provided to FAO by the French authorities to re-inforce the prevention and control of RVF (GCP/SNE/001/FRA). This allowed the organization of a first EQA dedicated to the RVFV by virus genome and antibodies detection in order to perform a first assessment of the diagnostic capacity of the laboratories involved in the RVF surveillance in the REMESA framework. This study reports the results from the inter-laboratory ring trials and provides details related to the diagnostic protocols used for RVF genome and antibodies detection.

## Materials and Methods

### Call for participation

An invitation letter was sent to the CVOs of the REMESA countries (Algeria, Egypt, France, Italy, Libya, Morocco, Mauritania, Portugal, Spain and Tunisia) in June 2012 by the Istituto Zooprofilattico Sperimentale dell’Abruzzo e del Molise– IZSAM. The organization of this trial, particularly with regard to the logistical and technical aspects, was presented and discussed to REMESA member countries during a preliminary meeting held in Tunis on 6 and 7 May 2013. A total of 10 laboratories involved in diagnostics of RVF infections from 6 different countries expressed their willingness to participate (3 from Algeria, 2 from France, 1 from Mauritania, 2 from Morocco, 1 from Spain and 1 from Tunisia). Six laboratories participated to both the viral genome detection by RT-PCR and the specific IgG and IgM antibodies detection. Four laboratories participated exclusively to the antibodies detection trial. The participation of each laboratory and the import of panels was officially requested by the organizer and authorized by the Chief Veterinary Officers of the participating countries.

### Specimen preparation

The study included preparation of two different test panels: i) sample panel for viral genome detection by RT-PCR, ii) sample panel for antibodies detection (IgG and IgM detection) by ELISA.

#### Samples for viral genome detection

For the molecular diagnosis of RVFV, each participant received a coded panel of 15 ruminant sera composed of 5 negative and 10 positive samples. The RVF field strain Namibia 2010 (N10) [20] was used to spike negative bovine serum to prepare the positive samples. Briefly, the viral strain was propagated on Vero cells (ATCC^®^ Manassas, USA), the supernatant collected and inactivated as follows: Tris–hydroxymethyl-aminomethane (Tris) 1 M pH 8.0 was added to the virus suspension and stirred for 10 min at room temperature. Beta-propiolactone diluted in PBS pH 7.4 was subsequently added to the virus suspension to a final dilution of the inactivation agent equal to 0.2% (v/v) [21]. Three consecutive passages on Vero cells, each followed by real-time quantitative RT-PCR (qRT-PCR) [10] excluded any residual infectivity. Five different dilutions (1:2, 1:10, 1:50, 1:100, 1:200) of the viral suspension were added to the RVFV seronegative bovine serum. The viral load was evaluated for each viral dilution by qRT-PCR [10]. The copy number of the RVFV genome was determined in each spiked sample by using serial dilutions of the linearized plasmid pEX-A (Eurofin Scientific, Luxemburg) containing the PCR target sequence. The viral load was defined as the mean value calculated from five replicates of each sample (Tables 1 and 2). Spiked samples were evaluated to assess their homogeneity and stability. Homogeneity was evaluated by testing 5 replicates of each sample. Stability was evaluated by testing three aliquots of each sample kept at room temperature and tested at different time intervals [t0, t1 (72 hours) t2 (7 days)]. Negative samples were prepared from a stock of RVFV seronegative serum. Aliquots of 300 μl were stored at −80°C and kept frozen until the shipment to individual laboratories.

**Table 1:** Results of the EQA for RVFV virus genome detection.

RVF strain	Namibia 2010														
ID Sample	1	2	3	4	5	6	7	8	9	10	11	12	13	14	15
Dilution	1:2	1:10	1:50	Neg	Neg	1:100	1:200	Neg	Neg	1:2	1:10	1:50	Neg	1:100	1:200
RVF copies/μl	10^6,1^	10^5,4^	10^4,8^			10^4,5^	10^4,2^			10^6,1^	10^5,4^	10^4,8^		10^4,5^	10^4,2^
**Lab n°**															
#1*	+	+	+	FP	-	+	+	-	-	+	+	+	-	+	+
#1a*	+	+	+	-	-	+	+	-	-	+	+	+	-	+	+
#2	+	+	+	-	-	Inc	+	-	-	+	Inc	Inc	-	FN	FN
#7	+	+	+	-	-	+	+	-	-	+	+	+	-	+	+
#8	+	+	+	-	-	+	+	-	-	+	+	+	-	+	+
#9	+	+	+	-	-	+	+	-	-	+	+	+	-	+	+
#10	+	+	+	-	-	+	+	-	-	+	+	+	-	+	+

Neg: RVFV seronegative bovine serum. + / -: samples identified as positive or negative by the participants. FP: false positive result. FN: false negative result. Inc: Inconclusive results.

* Laboratory providing multiple datasets

**Table 2:** Results of the EQA for RVFV virus genome detection, cycle threshold (Ct) values.

RVF strain	Namibia 2010														
ID Sample	1	2	3	4	5	6	7	8	9	10	11	12	13	14	15
Dilution	1:2	1:10	1:50	Neg	Neg	1:100	1:200	Neg	Neg	1:2	1:10	1:50	Neg	1:100	1:200
RVF copies/μl	10^6,1^	10^5,4^	10^4,8^			10^4,5^	10^4,2^			10^6,1^	10^5,4^	10^4,8^		10^4,5^	10^4,2^
**Lab n°**															
#1*	22,8	22,9	22,7	35,9	No Ct	23,8	24,6	No Ct	No Ct	24,5	21,8	24,7	No Ct	25,8	26,3
#1a*	24,5	22	26,9	No Ct	No Ct	26,6	27,7	No Ct	No Ct	25,1	22,1	24,8	No Ct	26,4	27,3
#2	31	32	34	No Ct	No Ct	39	35	No Ct	No Ct	31	38	39	No Ct	42	41
#7	21,6	23,9	26,0	No Ct	No Ct	27,7	27,7	No Ct	No Ct	21,7	22,7	25,3	No Ct	25,6	31,3
#8	26,8	25,7	28,6	40	40	29,6	29,9	40	40	27,9	26,9	29,2	40	29,6	30,4
#9	22.8	21.6	23.8	No Ct	No Ct	24.6	25.7	No Ct	No Ct	22.1	22.1	23.7	No Ct	24.7	25.7
#10	26,5	27,3	28,9	No Ct	No Ct	29,9	29,7	No Ct	No Ct	25,5	27,1	28,6	No Ct	27,1	29,4

Neg: RVFV seronegative bovine serum. No Ct: result above the threshold.

* Laboratory providing multiple datasets

#### Samples for antibodies detection by ELISA

For the serological diagnosis of RVF, each participant received a panel of 15 ruminant sera composed of 5 negative and 10 positive samples. The positive samples consisted of sera from domestic and wild ruminants: five samples were from RVFV vaccinated sheep (n = 4) and goats (n = 1) seropositive for IgG. Animals were vaccinated with the formalin inactivated Rift Valley fever virus commercially available from the Onderstepoort Biological Product (OBP, Onderstepoort, South Africa). Five samples were from 5 springboks (*Antidorcas marsupialis*) found RVF seropositive for both IgG and IgM according with the results of ELISAs ID Screen^®^ Rift Valley Fever Competition Multi-species and ID Screen^®^ Rift Valley Fever IgM Capture (IDvet, Grabels, France) for IgG and IgM detection respectively. Negative samples were prepared from a single bovine RVF seronegative serum (Gibco^®^ Fetal Bovine Serum, Life Technologies, Carlsbad, USA). To exclude the presence of any infectious viral particle, the samples were tested by RT-PCR [9] and heated at 56°C for three hours. The inactivation process was assessed as described above. Each set of samples was evaluated for homogeneity by testing 5 replicates with the above ELISAs. Stability was evaluated with the ELISA tests cited above by using the number of samples and the time intervals t0, t1 (72 hours) t2 (7 days).

### EQA details

The participants of the EQA were asked to analyze the panels by using the diagnostic procedures routinely used in their laboratories. They were also asked to provide details about the tests, namely the serological assay(s) used, the protocols for RT-PCR procedure, the manufacturer of the RT-PCR instrument and the chemicals for the RNA extraction.

### Statistical analysis

The results provided by each participant were classified as correct or incorrect on the basis of the *known* samples results in the panels. Results were analyzed by a Bayesian approach [22]. The Beta distribution was calculated and used to define the probability of each laboratory to give a correct result and the uncertainty of this estimate:

$$Beta(\alpha_{1},\alpha_{2})=\frac{x^{\alpha_{1}-1}{\left(1-x\right)}^{\alpha_{2}-1}}{\int_{0}^{1}t^{\alpha_{1}-1}{\left(1-t\right)}^{\alpha_{2}-1}dt}$$

where α_1_ = correct results +1; α _2_ = tested samples−correct results + l

### Ethics Statement

The serum samples distributed for the EQA were selected from the samples archive of the IZSAM, they were not collected specifically for this study. The collection of the samples was performed before the design of this study. The owner of the animals, the Central Veterinary Laboratory (CVL) of Windhoek (NA), kept them on pasture, free to graze grasses and forage found in the pasture and provided fresh water ad libitum. Following the notification of Rift Valley Fever (RVF) virus circulation in Namibia in 2010, CVL decided to protect the animals from the infection by administering an inactivated vaccine against RVF. Animals were bleed twice a month for approximately 6 months, in order to assess the sero-conversion. The IZSAM provided diagnostic support, in the frame of the international cooperation between the IZSAM and the Namibian Directorate of Veterinary Services, enforced by the Memorandum of Understanding signed the 16th December 2004 in Windhoek and the 24th January 2005 in Teramo, Italy. The activity was funded by Italian Ministry of Health (IZSAM01/10 RC) and only the CVL and IZSAM veterinarians and trained animal care personnel were allowed to manipulate and bleed the animals.

Serum samples from springbok were collected by jugular venopuncture during a disease surveillance program in wildlife in the Ethosa National Park (ENP) in Namibia. Animal darting, bleeding and post-operative care, as well as radiocollaring and tracking were carried out by ENP staff to minimize unnecessary stress and injury to the animals. Approval for sample collection was obtained by the Namibian Ministry of Environment and Tourism the 25th October 2010, (research/collection permit n° 1543/2010, 15/11/2010). The vaccination protocol as well as the collection of the springbok sera could not be submitted to an ethical body for approval, since Namibia did not apply animal testing regulations.

## Results

Ten laboratories participated in the EQA from 6 different countries (4 African and 2 European). The participants were (in alphabetical order by countries): i) Institut National de la Médecine Vétérinaire, Laboratoire Central Vétérinaire d'Alger, Algeria; ii) Institut National de la Médecine Vétérinaire, Laboratoire Vétérinaire Régional de Laghouat, Algeria; iii) Institut National de la Médecine Vétérinaire, Laboratoire Vétérinaire Régional de Tlemcen, Algeria; iv) Agence Nationale de Sécurité Sanitaire de l’Alimentation, de l’Environnement et du Travail (ANSES), Virology Unit, Laboratory of Lyon, France; v) Centre de Coopération Internationale en Recherche Agronomique pour le Développement (CIRAD), Montpellier, France; vi) Centre National d'Elevage et de Recherches Vétérinaires, laboratoire de Virologie, Nouakchott, Mauritania; vii) Office National de Sécurité Sanitaire des Produits Alimentaires (ONSSA), Laboratoire Régional d'Analyses et de Recherches d'Agadir, Morocco; viii) Office National de Sécurité Sanitaire des Produits Alimentaires (ONSSA), Laboratoire Régional d'Analyses et de Recherches de Casablanca, Morocco; ix) Centro de Investigación en Sanidad Animal (CISA-INIA), Laboratory of Emerging and Transboundary Diseases, Valdeolmos (Madrid), Spain; x) Institut de la Recherche Vétérinaire de Tunisie, Tunisia.

The laboratories in France (CIRAD and ANSES) and in Spain (CISA-INIA) performed the diagnostic test in BSL-3 facilities. The participating laboratories from African countries, lacking a BSL-3 facility, used biosafety cabinets and appropriate procedures and personal protective equipment to manipulate the samples.

### Virus genome detection

A total of 7 datasets were received from the 6 laboratories which participated to the EQA for the virus genome detection panel, including 1 double set from laboratory #1, which used two different methods (#1 and #1a). All labs used real-time RT-PCR assays. The primer sequences were from 2 different RT-PCR tests: 3 laboratories (#2, #7, #9) used the method developed by Drosten et al. [11] targeting the RVFV-M segment whereas 2 laboratories (#1, #8) used the methods published by Bird et al. [10] and LaBeaud et al. [23] (#10) using the same primers targeting the L segment. One laboratory (#1a) used an unpublished method developed by the Friedrich-Loeffler-Institut with the oligonucleotides published by Bird et al. [10] (Table 3). One single-base mismatch with the Namibia 2010 strain target sequence was present within the reverse primer of the method developed by Drosten et al. [11] and within the probe of the RT-PCR assay by Bird et al. [10]. RNA extraction techniques varied among participants: 4 carried out manual extraction with the commercial kits: (#1, #1a, #10) NucleoSpin^®^ RNA Virus (Macherey-NagelGmbH & Co. KG, Duren, Germany), (#2) PureLink^®^ Viral RNA/DNA Mini Kit (Life Technologies, Carlsbad, CA, USA), (#9) QIAamp^®^ Viral RNA Mini Kit (Qiagen, Venlo, Limburgo, Netherlands), while 2 laboratories relied on automated methods: (#7) BioSprint 15 workstation (Qiagen, Venlo, Limburgo, Netherlands) and (#8) Arrow Viral NA Kit (AutoGen Nordiag Holliston, MA, USA). Among the participating labs, 5 RT-PCR instruments were used: (#1, #1a) Swift^™^ Spectrum 48 Real Time Thermal Cycler (Esco Technologies,Hatboro, PA, USA), (#2) Applied Biosystems^®^ 7500 Real-Time PCR Systems (Life Technologies, Carlsbad, CA, USA), (#7, #10) Mx3005P qPCR (Agilent Technologies, Santa Clara, CA, USA), (#8) iQ5 (BioRad, Hercules, CA, USA), (#9) LightCycler^®^ 480 (Roche, Basilea, Switzerland). Reagents used for RT-PCR were: (#1, #8, #9) SuperScript^®^ III Platinum^®^ One-Step Quantitative RT-PCR System (Life Technologies, Carlsbad, CA, USA), (#1a) OneStep RT-PCR Kit (Qiagen, Venlo, Limburgo, Netherlands), (#7) AgPath-ID One-step RT-PCR kit (Life Technologies, Carlsbad, CA, USA), (#10) Sybr Green Brillant II (Agilent Technologies, Santa Clara, CA, USA) (Table 3).

**Table 3:** EQA for RVFV virus genome detection, RNA extraction and RT PCR instruments and methods.

	RNA		RT PCR		
	Extraction	Purification kit	Thermal Cycler	Reagents	Protocols
**Lab ID**					
#1*	Manual	NucleoSpin^®^ RNA Virus	Swift^™^ Spectrum 48 Real Time	SuperScript III Platinum One-Step qRT-PCR	Bird et al. 2007
#1a*	Manual	NucleoSpin^®^ RNA Virus	Swift^™^ Spectrum 48 Real Time	OneStep RT-PCR Kit	Unpublished**
#2	Manual	PureLink^®^ Viral RNA/DNA	Applied Biosystems^®^ 7500 Real-Time	na	Drosten et al, 2002
#7	Automated	BioSprint 15 workstation	Mx3005P qPCR	AgPath-ID One-step RT-PCR kit	Drosten et al, 2002
#8	Automated	Arrow Viral NA	iQ5	SuperScript III Platinum One-Step qRT-PCR	Bird et al. 2007
#9	Manual	QIAamp^®^ Viral RNA	LightCycler^®^ 480	SuperScript III Platinum One-Step qRT-PCR	Drosten et al, 2002
#10	Manual	NucleoSpin^®^ RNA Virus	Mx3005P qPCR	Sybr Green Brillant II	Labeaud et al. 2011

na = not available information

* Laboratory providing multiple datasets

**Friedrich-Loeffler-Institut (in-house) using primer sequences published by Bird et al. (2007)

**Fig 1:**
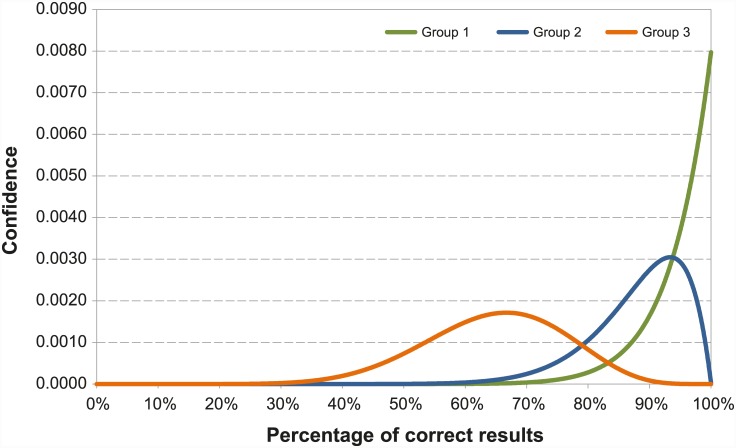
Distribution of the correct results of laboratories participating to EQA for RVFV genome detection. Group 1 represents laboratories which correctly classified 15 out of the 15 tested samples (#1a, #7, #8, #9, #10). Group 2 represents the laboratory failing to one test result (#1). Group 3 represents the laboratory, which misidentified 5 test results (#2).

Five out of 6 laboratories reported 100% of correct results (Table 1) Ct values provided by the laboratories are detailed in Table 2. The laboratory providing two different RT-PCR datasets produced 100% of correct results and one false-positive result respectively (Table 1). Laboratory #2 misidentified 2 positive samples as negative and 3 positive samples as doubtful (Tables 1 and 2). The laboratory failing one test result (#1) has a probability of giving a correct test result higher than 73.6% (Fig 1). For laboratories which correctly classified 15 out of 15 tested samples (labs # 1, 7, 8, 9, 10), the probability of giving a correct result was higher than 82.9%, with a confidence level of 95% (Fig 1).

### RVF antibodies detection (IgG/IgM)

To detect the RVF IgG antibodies, all participating laboratories used the commercial IgG-ELISA assay ID Screen^®^ Rift Valley Fever Competition multi-species (IDvet, Grabels, France). In addition one laboratory (#7) used the commercial INgezim FVR DR (Ingenasa, Madrid, Spain) (#7a) and confirmed the positive ELISA results with virus neutralization. A second laboratory (#9) tested two in-house IgG-ELISA assays based on: i) crude lysate of RVFV infected cell cultures (#9a) and ii) recombinant nucleocapsid protein of RVFV (#9b).

All laboratories used the same commercial kit for the serological detection of IgM, the ID Screen^®^ Rift Valley Fever IgM Capture (IDvet, Grabels, France). In addition one laboratory (#9a) tested the samples with an in-house IgM-capture ELISA assay based on the lysate and the supernatant of RVFV infected cell culture.

Thirteen datasets were received from the 10 laboratories participating to the RVF antibodies detection EQA panel.

Namely 8 labs used the same unique kit to detect IgG while 2 laboratories (#7 and #9) provided the results coming from 3 different methods. Nine out of 10 laboratories reported 100% of correct results. One laboratory, which used 2 different commercial IgG-ELISA reported 100% of correct results using one kit and all correct results except one false-positive when using a second assay (Table 4).

**Table 4:** Results of the EQA for RVF IgG antibodies detection.

**Sample description**																
ID Sample	1	2	3	4	5	6	7	8	9	10	11	12	13	14	15	
Species	Goat	Cattle	Wild rum	Wild rum	Wild rum	Cattle	Cattle	Sheep	Sheep	Cattle	Sheep	Wild rum	Wild rum	Sheep	Cattle	
IgG	Pos	Neg	Pos	Pos	Pos	Neg	Neg	Pos	Pos	Neg	Pos	Pos	Pos	Pos	Neg	ELISA
**Lab n°**																
#1	**+**	-	+	+	+	-	-	+	+	-	+	+	+	+	-	†
#2	**+**	-	+	+	+	-	-	+	+	-	+	+	+	+	-	†
#3	**+**	-	+	+	+	-	-	+	+	-	+	+	+	+	-	†
#4	**+**	-	+	+	+	-	-	+	+	-	+	+	+	+	-	†
#5	**+**	-	+	+	+	-	-	+	+	-	+	+	+	+	-	†
#6	**+**	-	+	+	+	-	-	+	+	-	+	+	+	+	-	†
#7*	**+**	FP	+	+	+	-	-	+	+	-	+	+	+	+	-	†
#7a*	**+**	-	+	+	+	-	-	+	+	-	+	+	+	+	-	‡
#8	**+**	-	+	+	+	-	-	+	+	-	+	+	+	+	-	†
#9*	**+**	-	+	+	+	-	-	+	+	-	+	+	+	+	-	†
#9a*	**+**	-	+	+	+	-	-	+	+	-	+	+	+	+	-	*in house assay* §
#9b*	**+**	-	+	+	+	-	-	+	+	-	+	+	+	+	-	*in house assay* § §
#10	**+**	-	+	+	+	-	-	+	+	-	+	+	+	+	-	(†)

Wild rum: wild ruminants (*Antidorcas marsupialis*). Pos: RVF seropositive status; Neg: RVF seronegative status. + /—: samples identified as positive or negative by the participants. FP: false positive result.

^*(†)*^IDvet: ID.Screen RVF competition multi- species kit.

^*(‡)*^Ingenasa: Ingezim FVR DR 13-FVR.K0.

^*§*^ *in house assay*: test based on crude cell lysate as antigen.

^*§ §*^ *in house assay*: test based on recombinant N protein as antigen.

* Laboratory providing multiple datasets

Regarding the RVF IgM detection, nine out of 10 laboratories reported 100% of correct results. One laboratory reported all correct results except one false-positive (Table 5).

**Table 5:** Results of the EQA for RVF IgM antibodies detection.

Sample description																
Sample	1	2	3	4	5	6	7	8	9	10	11	12	13	14	15	
Species	Goat	Cattle	Wild rum.	Wild rum	Wild rum	Cattle	Cattle	Sheep	Sheep	Cattle	Sheep	Wild rum	Wild rum	Sheep	Cattle	
IgM	Neg	Neg	Pos	Pos	Pos	Neg	Neg	Neg	Neg	Neg	Neg	Pos	Pos	Neg	Neg	ELISA
**Lab n°**																
#1	-	-	+	+	+	-	-	-	-	-	-	+	+	-	-	†
#2	-	-	+	+	+	-	-	-	-	-	-	+	+	-	-	†
#3	-	-	+	+	+	FP	-	-	-	-	-	+	+	-	-	†
#4	-	-	+	+	+	-	-	-	-	-	-	+	+	-	-	†
#5	-	-	+	+	+	-	-	-	-	-	-	+	+	-	-	†
#6	-	-	+	+	+	-	-	-	-	-	-	+	+	-	-	†
#7	-	-	+	+	+	-	-	-	-	-	-	+	+	-	-	†
#8	-	-	+	+	+	-	-	-	-	-	-	+	+	-	-	†
#9*	-	-	+	+	+	-	-	-	-	-	-	+	+	-	-	†
#9a*	-	-	+	+	+	-	-	-	-	-	-	+	+	-	-	§
#10	-	-	+	+	+	-	-	-	-	-	-	+	+	-	-	†

Wild rum: wild ruminants (*Antidorcasmarsupialis*). Pos: RVF seropositive status; Neg: RVF seronegative status. + /—: samples identified as positive or negative by the participants. FP: false positive

^*†*^IDvet: ID. Screen RVF IgM Capture

^*§*^ *In-house assay*: test based on crude cell lysate as antigen

* Laboratory providing multiple datasets

**Fig 2:**
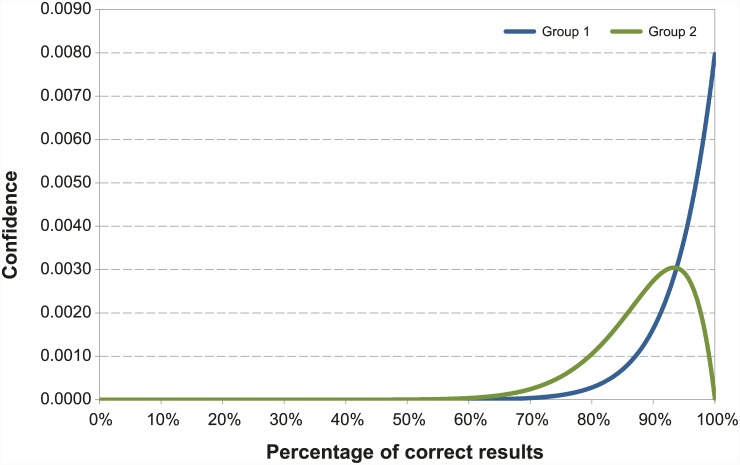
Distribution of the correct results of laboratories participating to the EQAs for RVF antibodies detection (IgG and IgM). Group 1 includes laboratories, which correctly classified 15 out of the 15 tested samples, Group 2 represents the laboratory failing to one test result. For IgG EQA group 1 includes the laboratories: #1, #2, #3, #4, #5, #6, #7a, #8, #9, #9a, #9b, #10, while in group 2 #7. For IgM EQA group 1 includes #1, #2, #4, #5, #6, #7, #7a, #8, #9, #9a, #9b, #10 and group 2 #3.

In both trials aiming to detect RVF specific IgG and IgM, the laboratories failing in one diagnostic test have a probability of giving a correct test result higher than 73.6% with a confidence level of 95%. The laboratories which correctly classified 15 out of 15 tested samples have more than 82.9% probability of giving a correct test result a confidence level of 95% (Fig 2).

## Discussion

In May 2013, during the REMESA meeting held in Tunis, the IZSAM, in collaboration with FAO and OIE, presented the first planned EQA for the diagnosis of RVF in animals. From October to December 2013 two panels, a first one addressing the RVFV genome detection, and a second one addressing the RVF antibodies detection, were prepared and shipped to the participating laboratories. Results from participants were received from November 2013 to February 2014. In April 2014 the final report of the ring trial was sent to all participants and organizers.

The EQA aimed to get a preliminary evaluation of the diagnostic capacities of the participating laboratories for their ability to detect RVF antibodies and RVFV genome from serum samples. The tests were intended as qualitative and, therefore, the accuracy of the results were assessed on the basis of the “positive”, “negative” and/or “doubtful” results received from the participating laboratories.

The panel for the virus genome detection consisted of 15 samples containing various concentrations of the Namibia 2010 strain [20]. Considering the limited genetic diversity of RVFV strains in nature (4% differences for the nucleotide sequence and 1% for the amino acid sequence) [7] it was considered enough to include a single, well characterized, virus strain in the panel dedicated to the virus genome detection. The panel dedicated to RVF antibodies detection consisted of 15 samples including domestic sheep and goats and springbok (*Antidorcas marsupialis*) sera with different levels of RVF specific IgG and/or IgM. Both panels represented a comprehensive proficiency test to assess the laboratories capacity to detect either antibodies or viral genome in several animal species. Proficiency testing on molecular diagnosis of RVF reported 100% concordant results in 5 of the 7 dataset submitted. One laboratory (# 1) reported 1 false positive result with one RT-PCR assay [10], while the same sample was correctly reported as negative using a second (unpublished) protocol. One laboratory (# 2) reported 2 false negatives and uncertain results for 3 positive samples with the assay described by Drosten et al [11], though the same technique was used by 2 different labs (# 7, # 9) producing the expected results. Analyzing the RT-PCR performances, outside lab #2, which was below the expected level of sensitivity, it was possible to observe some variability in Ct values ranging from 1 to 4 Ct. The quality of purified RNA, primers, probes, PCR instruments and commercial real-time RT-PCR kits used could have been responsible for the variation in sensitivity observed in the participating labs. Furthermore the conditions applied to RT-PCR, the concentration of the reaction components and time and temperature parameters, should be carefully optimized to achieve efficient amplification of the specific target. All laboratories participating to this study reported the use of real time RT-PCR technique. This confirms that the use of real time RT-PCR has remarkably expanded, replacing conventional gel-based RT-PCR method, although real-time requires expensive equipment and trained personnel. In similar ring trials for RVF molecular detection [24], authors reported the use of other conventional techniques, e.g. RT-nested PCR, RT Loop-mediated isothermal amplification (RT-LAMP) and recombinase polymerase amplification (RPA) together with real-time RT-PCR for the detection of RVFV nucleic acids.

All RT-PCR assays showed optimal performances, providing accurate determination of positive and negative results. The level of sensitivity using RT-PCR achieved by the participants to this EQA is not different from that reported in the study of Escadafal *et al*. [24].

A total of 4 commercially available ELISA and 3 in-house assays were used by the participating laboratories for antibodies detection. All participants used IDvet ELISA kits (ID Screen^®^ Rift Valley Fever Competition Multi-species for IgG detection and ID Screen^®^ Rift Valley Fever IgM Capture for IgM detection). One laboratory (#7) used in parallel the commercial INgezim FVR DR (Ingenasa, Madrid, Spain) for IgG detection. One laboratory (#9) tested 2 in-house ELISA assays for IgG detection, based on recombinant nucleocapsid protein of RVFV and crude lysate of RVFV infected cell cultures respectively. The same laboratory evaluated in-house ELISA assay for IgM using infected cell lysate as antigen. Furthermore one laboratory (#7) confirmed the ELISA positive samples with virus neutralization. Overall, the in-house and commercial assays used in the EQA performed with no variation. The EQA presented here provided a good overview on the laboratory capacities for the diagnosis of RVF in animals in the Western Mediterranean Region, showing that the participating laboratories were able to perform the tests and provide the results in time. The use of a limited set of diagnostic assays indicated that harmonized procedures are already being applied by the participating laboratories, allowing the comparison of results and thus indicating that an efficient regional surveillance system, which is one of the objectives of REMESA, may be put in place. Furthermore the combination in the same EQA of molecular and serological techniques, which allows a complementary diagnostic approach to RVF, is particularly important for diagnosis and monitoring mainly in countries where RVFV is not endemic.

Otherwise, the use of the same tests by all participants may limit the possibility to recognize the emergence of unusual virus strains evoking different immune responses [25].Therefore the use of a wider set of diagnostic methods should be encouraged.

To guarantee a constant high quality level of RVF diagnosis in the region and to ensure the reliability of the diagnostic results we recommend conducting EQA studies on a regular basis.
